# Nonalcoholic Fatty Liver Disease Increases the Risk of Lung Abscess: Findings from a Nationwide Cohort Study

**DOI:** 10.3390/jcm14020542

**Published:** 2025-01-16

**Authors:** Eunso Lee, Jungok Kim, Sun-Young Yoon

**Affiliations:** 1 Division of Allergy and Pulmonology, Department of Internal Medicine, Chungnam National University Sejong Hospital, Sejong-si 30099, Republic of Korea; 2 Division of Infectious Diseases, Department of Internal Medicine, Chungnam National University Sejong Hospital, Sejong-si 30099, Republic of Korea; 3 Department of Medicine, Chungnam National University School of Medicine, Deajeon 35015, Republic of Korea

**Keywords:** nonalcoholic fatty liver disease, lung abscess, fatty liver index, community-acquired pneumonia

## Abstract

**Objectives:** This study aimed to investigate the association between nonalcoholic fatty liver disease (NAFLD), assessed by the Fatty Liver Index (FLI), and the occurrence of lung abscess within a large population-based cohort. **Method:** We conducted a nationwide retrospective study using data from 367,930 subjects who underwent National Health check-ups between 2009 and 2018. Cox proportional hazards regression was performed to evaluate the association between the FLI and the incidence of lung abscess and community-acquired pneumonia (CAP) after adjusting for age, sex, and relevant covariates. **Results:** Among the study population, 455 (0.12%) and 44,934 (12.2%) patients were diagnosed with lung abscesses and CAP, respectively. The cumulative incidence of lung abscess was higher in individuals with elevated FLI values (FLI < 30, 0.10%; 30 ≤ FLI < 60, 0.16%; FLI ≥ 60, 0.18%; *p* < 0.001), whereas the incidence of CAP decreased across FLI groups (FLI < 30, 12.4%; 30 ≤ FLI < 60, 12.3%; FLI ≥ 60, 11.0%; *p* < 0.001). After adjusting for covariates, the risk of lung abscess significantly increased in the 30 ≤ FLI < 60 (Hazard ratio (HR) = 1.26; 95% confidence interval (CI), 0.95–1.68; *p* = 0.115) and the FLI ≥ 60 (HR = 1.67; 95% CI, 1.37–2.29; *p* < 0.001) groups, although the risk of CAP was relatively small in both groups (30 ≤ FLI < 60; HR = 1.06; 95% CI, 1.03–1.09; *p* < 0.001) (FLI ≥ 60; HR = 1.13; 95% CI, 1.08–1.12; *p* < 0.001). **Conclusions:** Our study provides compelling evidence supporting a potential link between NAFLD, as measured by FLI, and the incidence of lung abscess. These findings suggest the importance of vigilant monitoring of respiratory health in patients with NAFLD and emphasise the need for early detection of possible complications.

## 1. Introduction

Nonalcoholic fatty liver disease (NAFLD) is a significant public health concern worldwide, paralleling the global surge in obesity, sedentary lifestyles, and unhealthy dietary habits [1]. Its prevalence, diagnosed using imaging, stands at approximately 25% of the general population [2]. NAFLD comprises a spectrum of liver conditions, ranging from simple hepatic steatosis with or without mild inflammation (nonalcoholic fatty liver, NAFL) to more severe forms of nonalcoholic steatohepatitis and advanced fibrosis, with the potential to progress to cirrhosis and hepatocellular carcinoma [3]. Its association with metabolic disorders, such as diabetes and cardiovascular diseases, is widely acknowledged [4]. However, evidence indicates that NAFLD may increase the risk of chronic inflammation or infections [5]. Although the precise pathogenesis of NAFLD during infection or inflammation remains incompletely understood, damage to the liver architecture, which is vital for host defence against infectious diseases, may gradually impair the immune response and potentially increase susceptibility to infection among individuals with NAFLD [6]. Previous studies have reported a relationship between NAFLD and respiratory infections, including community-acquired pneumonia (CAP) and specific pneumonia due to coronavirus disease (COVID-19), which are associated with poor outcomes such as higher mortality rates and prolonged hospital stays [7,8,9,10]. However, to our knowledge, no study has addressed the association between NAFLD and lung abscesses.

Lower respiratory tract infections are a leading cause of mortality and result in substantial healthcare costs [11]. Lung abscess, a serious respiratory infection, is characterised by the formation of a localised collection of pus within the lung parenchyma, often resulting from aspiration pneumonia, bronchial obstruction, or hematogenous spread of the infection [12]. Despite advancements in antimicrobial therapy, lung abscesses still have poor outcomes, particularly in vulnerable populations, such as individuals with underlying comorbidities [13]. While individuals with underlying conditions prone to aspiration face the risk of developing lung abscesses, they can also occur in healthy adults with no discernible medical history, particularly in individuals with poor oral hygiene. These cases are often linked to pharyngeal aspiration during deep sleep, associated primarily with anaerobic bacteria and microaerophilic streptococci, and less commonly with gram-negative bacilli [14,15]. Although several risk factors for lung abscesses have been identified, including smoking, alcohol abuse, and immunosuppression, the role of NAFLD in the development of lung abscesses has not been examined.

Given the systemic nature of NAFLD and its association with chronic inflammation, it may increase the risk of respiratory infections such as lung abscesses. Therefore, the objective of this study was to investigate the association between NAFLD, as assessed using the fatty liver index (FLI), and the incidence of lung abscesses in a large population-based cohort. Additionally, we sought to evaluate the relationship between NAFLD and CAP to comprehensively understand the systemic implications of NAFLD on respiratory health. By clarifying the link between NAFLD and lung abscess as well as CAP, this study aimed to assist in the development of strategies for the prevention of respiratory infections in individuals with NAFLD.

## 2. Material and Methods

### 2.1. Data Sources

We conducted a nationwide retrospective cohort study utilising data extracted from the National Health Insurance Service-National Sample Cohort 2.2 (NHIS-NSC 2.2), covering the period from 1 January 2002 to 31 December 2019. The national health screening program in South Korea was launched by the government in 1980 to promote public health through early detection and prevention of diseases [16]. The program offers biennial health check-ups to all citizens, including general health and cancer screenings. These check-ups encompass blood tests, chest X-rays, physical examinations, medical history reviews, and questionnaires on health-related behaviors. The total population was stratified into 2142 categories based on gender, age, region, eligibility status, and income level, with 2.2% of each category randomly selected [17]. With approximately 97% of Korea’s total population covered by the National Health Insurance, the NHIS-NSC 2.2 dataset is widely regarded as a representative of the entire Korean population [18]. Our sample comprised about 2% of the population and serves as a standardised sample provided by the NHIS. This database encompasses a wide array of information, including patient demographics, clinical information, 10th revision International Classification of Diseases (ICD-10) codes, medical claims, including diagnosis statements by ICD-10, treatment records, healthcare utilisation data, and health screening results. Furthermore, it incorporates mortality data such as the date and cause of death, sourced from the death registration database of Statistics Korea [17].

The Institutional Review Board of the Chungnam National University Sejong Hospital approved this study (IRB No. 2023-10-011). This study complied with the Declaration of Helsinki in 1964. Informed consent was waived because of the retrospective design; this study excluded any personal identification information, and the Institutional Review Board approved the waiver of informed consent. All authors had access to the study data and reviewed and approved the final manuscript.

### 2.2. Study Population

We included data from all individuals aged 20 years and above who underwent national health screening between January 2009 and December 2018. The initial health examination was designated as the index check-up, and the year in which the index check-up data were collected was regarded as the index year. To ensure an impartial assessment of the association between NAFLD and both lung abscess and CAP, individuals were excluded if they had (a) diagnoses of chronic liver disease, chronic pulmonary disease, autoimmune disease, cerebrovascular disease, malignant neoplasm, neuromuscular disease, or immunodeficiency disease within two years preceding the index year; (b) documented prescriptions for lipid-lowering, oral hypoglycaemic, or antihypertensive medications within two years prior to the index year; or (c) incomplete or missing data during the specified period. Diagnostic codes used in the analysis are listed in the web-only Supplementary Table S1.

### 2.3. Assessment of NAFLD

We employed the FLI as our assessment tool for NAFLD due to its non-invasive nature and well-established validity [19,20]. The FLI’s accuracy, with Area Under the Receiver Operator Characteristic Curve (AUROC) values ranging between 0.82 and 0.84 in both Western and Asian populations for predicting NAFLD, affirms its reliability [21]. Considering the potential limitations in recruitment based solely on ICD codes for identifying fatty liver cases, as well as constraints in accessing the results of liver biopsies or imaging techniques, such as abdominal ultrasound or computed tomography (CT), we opted for FLI as a dependable alternative. FLI measurements were available from the dataset derived for the cohort study and incorporated parameters such as body mass index (BMI), waist circumference (WC), triglycerides (TG), and gamma-glutamyl transferase (GGT). The FLI score was determined using the following equation [22]:

$$\mathrm{FLI}=\frac{e^{0.953In\left(TG\right)+0.139\times BMI+0.718\times In\left(GGT\right)+0.053\times WC-15.745}}{1+e^{0.9533In\left(TG\right)+0.139\times BMI+0.718\times In\left(GGT\right)+0.053\times WC-15.745}}\;*\;100$$

The FLI score extends from 0 to 100, with the original study recommending a cut-off value of ≥60 for the diagnosis of fatty liver, which yielded a positive likelihood ratio of 4.3. In this study, subjects were categorised into three groups based on the FLI score: 0 ≤ FLI < 30, 30 ≤ FLI < 60, and FLI ≥ 60, in accordance with previous research findings [22].

### 2.4. Outcomes

Incidence data for lung abscess and CAP were obtained using ICD-10 codes (lung abscess, J85.x-J86.x; CAP, J10.x-J18.x) and aligned with the dates of inpatient or outpatient clinic visits. To facilitate comprehensive evaluation, data on intensive care unit (ICU) admission and length of hospital stays were collected for severe cases. These cases were censored upon detection of lung abscess and CAP, as recorded by claims reflecting the patient’s hospitalisation or ICU stay, along with the presence of ICD-10 codes for lung abscess and CAP. Data were censored until individuals exited the NHIS owing to death or emigration.

### 2.5. Statistical Analysis

Continuous variables are presented as the mean ± standard deviation (SD), and categorical variables are expressed as the number of cases and percentage. Differences between the FLI groups were compared using the chi-square test for categorical variables or one-way analysis of variance (ANOVA) for continuous variables. The incidences of lung abscesses and CAP were measured as the number of events. The lengths of hospital and ICU stays were calculated as the number of events per 1000 person-years (PYs). Cumulative incidence rates were compared among the FLI groups using Kaplan–Meier curves and log-rank tests. Univariate and multivariate Cox proportional hazards regression analyses were performed to assess the influence of the FLI on the development of lung abscess and CAP while controlling for key covariates. We computed multivariable-adjusted hazard ratios (HRs) for two models: Model 1 was adjusted for age and sex, and Model 2 was further adjusted for age, sex, and covariates associated with the occurrence of lung abscess and CAP, exhibiting statistical significance at *p* < 0.1. This approach allowed for the consideration of confounding factors and enhanced the association between the FLI and outcomes. The results of the Cox regression analysis are expressed as hazard ratios (HRs) and corresponding 95% confidence intervals (CIs). Additionally, HRs were analysed for lung abscess and CAP within each subgroup based on biologically plausible factors to assess the independent effect of the FLI using forest plots. A two-sided *p* value of <0.05 was considered statistically significant. Statistical analyses were conducted using the R software, version 3.3.3 (R Foundation for Statistical Computing, Vienna, Austria; www.r-project.org; accessed on 1 September 2024).

## 3. Results

### 3.1. Baseline Characteristics

**Figure 1:**
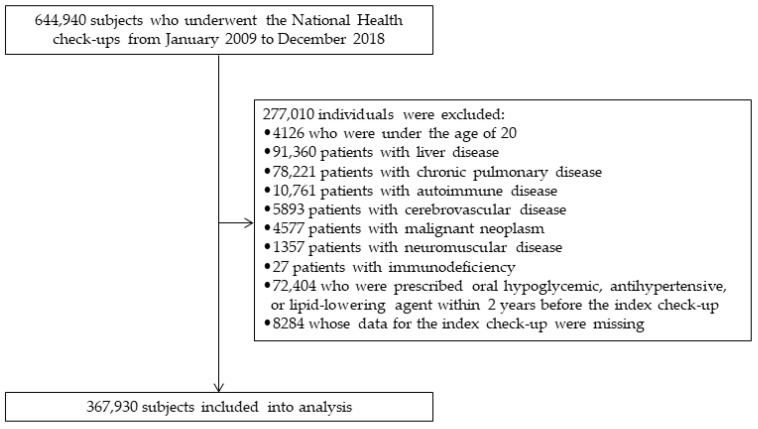
Enrollment of the study population.

From a pool of 644,940 individuals who underwent National Health check-ups, data from 367,930 were included in the analysis after exclusion (Figure 1). Table 1 presents the demographics and laboratory findings of the FLI group. Compared to the higher FLI groups, the FLI < 30 group exhibited a predominance of young individuals and females. As expected, higher FLI values correlated with elevated BMI, WC, and blood pressure and a higher prevalence of alcohol consumption. Laboratory parameters, such as TG, GGT, aspartate aminotransferase (AST), alanine aminotransferase (ALT), and fasting glucose levels, also increased with increasing FLI.

### 3.2. FLI and the Incidence of Lung Abscess and CAP

**Figure 2:**
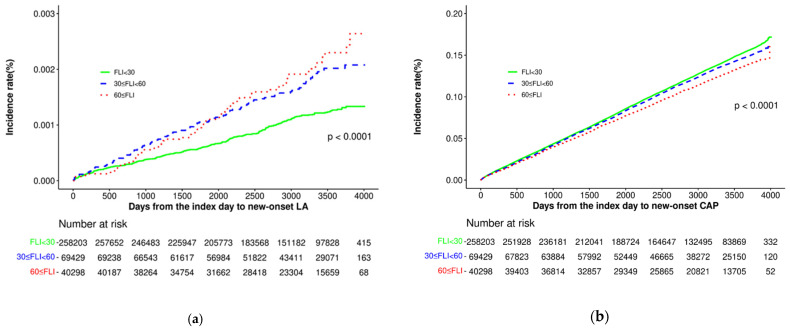
Cumulative incidence of lung abscess (**a**) and CAP (**b**) according to the FLI groups.

Throughout the entire cohort, 455 (0.12%) individuals developed lung abscess, while 44,934 (12.2%) developed CAP, with median follow-up periods of 9.1 and 8.4 years, respectively. The cumulative incidence of lung abscesses and CAP in the FLI group is shown in Figure 2. Compared to individuals with lower FLIs, those with higher FLIs experienced lung abscess more frequently (FLI < 30, 0.10% (266/258,203); 30 ≤ FLI < 60, 0.16% (116/69,429); FLI ≥ 60, 0.18% (73/40,298); *p* < 0.001) (Table 2). However, the incidence of CAP among FLI groups decreased (FLI < 30, 12.4% (31,988/258,203); 30 ≤ FLI < 60, 12.3% (8510/69,429); FLI ≥ 60, 11.0% (4436/40,298); *p* < 0.001).

No differences were noted in 30-day mortality, ICU admission, or length of hospital stay among the FLI groups upon diagnosis of lung abscess and CAP, although individuals with the highest FLI values had prolonged hospital stays (see web-only Supplementary Table S2).

### 3.3. Association Between FLI and Lung Abscess and CAP

In univariate Cox regression, with FLI < 30 as the reference category, the risk of lung abscess increased in the 30 ≤ FLI < 60, with an HR of 1.58 (95% CI, 1.27–1.96; *p* < 0.001), and FLI ≥ 60, with an HR of 1.77 (95% CI, 1.37–2.29; *p* < 0.001), groups (Table 2). Conversely, the risk of CAP slightly decreased in the 30 ≤ FLI < 60, with an HR of 0.96 (95% CI, 0.94–0.98; *p* < 0.001), and FLI ≥ 60, with an HR of 0.89 (95% CI, 0.86–0.91; *p* < 0.001), groups. To ascertain the independent effect of the FLI, adjustments were made to the covariates. After adjustment by age and male sex (model 1) according to FLI groups, the HR for lung abscess was 1.00 (95% CI, 0.80–1.25; *p* = 0.983) for 30 ≤ FLI < 60 and 1.23 (95% CI, 0.94–1.60; *p* = 0.129) for FLI ≥ 60. The HR for CAP did not differ significantly between the FLI groups. Further adjustment by adding significant covariates (model 2) revealed that FLI ≥ 60 was significantly associated with a 1.67-fold increased risk of lung abscess prevalence (95% CI, 1.06–2.63; *p* = 0.028), while the association was relatively small for CAP (HR 1.13; 95% CI, 1.08–1.12; *p* < 0.001).

For CAP, Model 2 included SBP, DBP, smoking, alcohol consumption, activity level, AST, ALT, haemoglobin, fasting glucose, low-density lipoprotein (LDL), and creatinine as covariates. Abbreviations: FLI, fatty liver index; CAP, community-acquired pneumonia; HR, hazard ratio; CI, confidence interval

Regarding other risk factors for lung abscess, male sex and smoking exhibited the highest associations, whereas additional factors, such as older age, elevated blood pressure, and FLI measurements, were also identified in the unadjusted Cox regression (see web-only Supplementary Table S3). Similarly, risk factors for CAP observed through the unadjusted analysis included these variables with the addition of higher fasting glucose levels.

**Figure 3:**
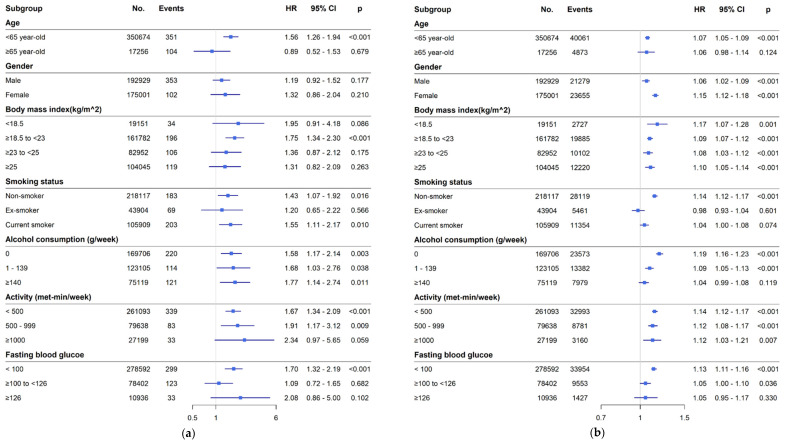
Forest plot of hazard ratios for (**a**) lung abscess and (**b**) CAP according to clinical subgroups. Abbreviation: HR, hazard ratio; CI, confidence interval; CAP, community acquired pneumonia.

The forest plot in Figure 3 shows the HRs for the occurrence of lung abscess and CAP in each clinical subgroup. Each adjusted HR for the respective subgroups presented a detailed analysis of FLI’s association with these outcomes across patient characteristics. The FLI was identified as a remarkable determinant of lung abscess, exhibiting an independent association unaffected by alcohol consumption and physical activity levels. Additionally, it was closely associated with incident lung abscesses in younger participants (<65 years), individuals with lower BMI (<23 kg/m^2^), and those with lower fasting blood glucose levels (<100 mg/dL) (Figure 3a). In the CAP group, the FLI was identified as a risk factor for developing CAP, independent of sex, BMI, alcohol consumption, and physical activity levels (Figure 3b).

## 4. Discussion

This study offers valuable insights into the association between NAFLD and the risks of lung abscesses and CAP. NAFLD, as measured using the FLI, was independently associated with an increased risk of lung abscess development. Additionally, we observed a strong effect of NAFLD on the risk of CAP after adjusting for covariates. Notably, this study is the first investigation to assess the relationship between FLI and lung abscess risk within a large population cohort, leading to its reliability and representation of the broader Korean adult population.

Previous studies have consistently highlighted the association between NAFLD and various infectious diseases, including CAP, gastrointestinal infections, recurrent bacterial infections, and specific pathogens such as *Clostridioides difficile*, *Helicobacter pylori*, COVID-19, hepatitis B virus (HBV), hepatitis C virus (HCV), and human immunodeficiency virus (HIV) [5,23,24,25,26,27,28]. Recent large-scale data on patients with biopsy-proven NAFLD have shown a heightened risk of assorted infections requiring hospitalisation compared with the general population [29]. Furthermore, the risk of infection appears to increase with the worsening histological severity of NAFLD, ranging from a 1.64-fold increase in simple steatosis to a 2.32-fold increase in cirrhosis. In a study focusing on patients with CAP admitted to hospitals, NAFLD, defined by imaging, was markedly associated with a 2.5-fold increase in CAP risk [7]. In our results, the risk of lung abscess and CAP was 1.67 and 1.13, respectively, in patients with FLI ≥ 60. Our study contributes to this body of evidence by demonstrating a correlation between high FLI scores, a well-validated measure of NAFLD, and the occurrence of lung abscesses and CAP in healthy adults at a nationwide level, after controlling for potential confounding variables. This finding suggests that NAFLD is associated with an increased risk of lung abscesses.

The mechanisms underlying the susceptibility of patients with NAFLD to infections are not fully understood; however, several pathways may be involved. Steatosis, a hallmark feature of NAFLD, disrupts the sinusoid microcirculation in the liver, disturbing hepatic microbial clearance. Additionally, an imbalance between fat storage and utilisation can promote a proinflammatory state in visceral adipose tissue, inducing insulin resistance and subsequent immune dysfunction [30]. Inflammatory macrophages, prominent in NAFLD, not only drive excessive hepatic inflammation and injury but also participate in chronic inflammatory processes in other tissues, such as adipose tissue or gut, where increased intestinal permeability may foster bacterial overgrowth and translocation [31]. Furthermore, the interplay and interdependence between the liver and other organs may exacerbate the risk of infection. Hepatic dysfunction could perturb inflammatory processes in the lungs by altering the innate immune signals from the liver during pneumonia [32]. This scenario might postulate a functional component of hepatic responses during respiratory infections, especially in the context of chronic liver conditions such as NAFLD, which could undermine the immune response.

Alterations in the microbiome composition in patients with NAFLD may signify another relevant mechanism of infection susceptibility. Dysbiosis of the gut microbiota is considered a possible contributor to the pathogenesis of NAFLD [33]. Bacterial signatures within the gut microbiome show heterogeneity across different stages of NAFLD progression, often accompanied by elevated levels of anaerobic bacteria [34]. Lung abscess commonly arises from the aspiration of oropharyngeal secretions, where anaerobic bacteria tend to be the predominant pathogens [35,36]. The microbiomes of the oropharynx harbour the causal pathogens of lung abscesses and act as endogenous reservoirs for the gut microbiota. Although the connection between microbiome alterations and respiratory infections has not been well studied in patients with NAFLD, previous research has described an association between NAFLD and periodontal diseases [37]. Consequently, an altered microbiome composition, specifically the increased presence of anaerobes, may be implicated in the pathogenesis of lung abscesses in individuals with NAFLD.

We observed that the impact of FLI on the occurrence of lung abscesses was pronounced in younger individuals and those with lower BMI in the subgroup analysis. To confirm the independent correlation between lung abscesses and NAFLD, we excluded factors that could affect the development of NAFLD and lung abscesses such as underlying chronic diseases and metabolic disorders. Given that these conditions occur more frequently in older adults, the exclusion of these patient groups resulted in a higher relative association of lung abscess occurrence in the younger age groups. Regarding its association with low BMI, the concept of lean or non-obese NAFLD has garnered attention owing to its unfavourable outcomes [38]. Although both lean and obese individuals with NAFLD share several metabolic abnormalities, they diverge in terms of genetic predisposition, body composition, gut microbiota, and susceptibility to environmental factors [39]. Considering the increased risk of infections in underweight patients and the high prevalence of non-obese NAFLD in Asian Countries, it is conceivable that a low BMI could have increased the risk of respiratory infections in our study population [38,40]. Further assessment of these issues is warranted to understand the complex traits of NAFLD.

NAFLD plays a pivotal role in the host defence against microorganisms and affects the progression and outcome of infections. Patients with NAFLD experience a more complex infection course and higher fatality rates than individuals with a healthy liver [5]. NAFLD has been associated with elevated rates of sepsis and hospital mortality, frequently necessitating critical care support, indicative of a more challenging infection trajectory [41]. Patients with NAFLD, particularly those with advanced hepatic fibrosis, are at higher risk of fatal outcomes from CAP [8]. However, our study found no correlation between higher FLI values and 30-day mortality, ICU stay in severe cases, or increased healthcare utilisation such as hospitalisation.

This observation may be attributed to the early-stage cases represented in our study population, as indicated by FLI scores before diagnosis or progression to advanced hepatic fibrosis. Furthermore, our study excluded individuals with comorbidities associated with metabolic syndrome such as diabetes and hypertension, which could influence infection outcomes. Notably, the length of hospital stay was considerably prolonged in patients with high FLI scores. This may imply that patients with NAFLD incur higher healthcare costs owing to longer hospitalisation, although the reasons for prolonged hospitalisation were not explained in our study.

Although our study investigated the association between NAFLD and lung abscesses, some limitations should be acknowledged. First, the reliance on data from a nationwide cohort introduces potential selection bias, as this may exclude individuals who do not undergo national health screening. Additionally, the use of administrative data for disease ascertainment based on diagnostic coding could lead to the misclassification or underestimation of outcomes. Moreover, the absence of information on certain variables such as environmental exposure hinders the ability to thoroughly explore potential confounders and effect modifiers. Second, the retrospective design of our study makes it susceptible to inherent biases and confounding factors that may not have been fully accounted for. Despite adjusting for numerous covariates, the possibility of residual confounding remains because unmeasured or imperfectly measured factors may influence the associations. Third, although the FLI is a validated tool for assessing NAFLD, it may not accurately capture all cases and lacks the ability to distinguish between the different stages of NAFLD. However, the FLI has been validated in both Asian and western populations and is commonly used as a proxy for NAFLD assessment [19,21]. Fourth, to reduce the impact of underlying lung conditions on respiratory infections, we excluded patients diagnosed with chronic respiratory diseases within two years prior to the analysis. However, we were unable to exclude patients newly diagnosed during the follow-up period after inclusion in the analysis. While the characteristics of big data research involving a large number of patients may somewhat mitigate this limitation, as it could have influenced the analysis results. Further research is needed to clarify this aspect. Fifth, pulmonary function data were not assessed in the cohort population. This correlation might be influenced by factors such as smoking history, which is more prevalent among individuals with NAFLD [42]. Patients with NAFLD may indeed have worse baseline pulmonary function [43]. Future research could explore the relationship between pulmonary function and respiratory infections in NAFLD patients. Finally, the generalisability of our findings may be confined primarily to Koreans undergoing national health screening, and the retrospective design precludes the establishment of causality. Future prospective studies involving other national populations are required to corroborate these findings.

To conclusion, our nationwide cohort study provides compelling evidence of an association between NAFLD and increased risk of lung abscesses. This study highlights the significance of NAFLD as a risk factor for respiratory infections, emphasising the importance of early detection and management of NAFLD to mitigate the associated complications. Given the ease of calculating the FLI in clinical settings, attention should be directed towards individuals with elevated FLI scores. Clinicians treating patients with NAFLD should be aware of the potential risk of developing lung abscesses. These findings underscore the critical need to recognise the impact of NAFLD on respiratory health and advocate further research to elucidate the underlying mechanisms. Future studies should investigate the specific pathogens associated with lung abscesses in NAFLD patients to better understand the potential immune dysfunction occurring in this population and to clarify the mechanisms underlying the increased risk of respiratory infections. By addressing these knowledge gaps, we can enrich our understanding of NAFLD’s systemic implications and devise targeted interventions to alleviate the burden of respiratory infections in affected individuals.

## Figures and Tables

**Table 1:** Baseline characteristics of the study cohort according to FLI groups.

Variables	FLI < 30 (*n* = 258,203)	30 ≤ FLI < 60 (*n* = 69,429)	FLI ≥ 60 (*n* = 42,298)	*p*
Age (years)	40.5 ± 13.0	44.2 ± 12.2	42.0 ± 10.9	<0.001
Male	103,891 (40.2)	53,680 (77.3)	35,358 (87.7)	<0.001
Height (cm)	163.8 ± 8.8	167.9 ± 8.7	170.2 ± 7.9	<0.001
Weight (kg)	59.1 ± 9.2	72.0 ± 9.0	81.6 ± 12.0	<0.001
BMI (kg/m^2^)	22.0 ± 2.5	25.5 ± 2.4	28.1 ± 3.4	<0.001
Waist circumference (cm)	74.5 ± 7.1	85.6 ± 5.4	92.4 ± 13.3	<0.001
SBP (mmHg)	116.8 ± 13.5	125.0 ± 13.9	129.6 ± 14.7	<0.001
DBP (mmHg)	72.9 ± 9.3	78.4 ± 9.7	81.9 ± 10.4	<0.001
Smoking				<0.001
Non-smoker	177,835 (68.9)	28,529 (41.1)	11,753 (29.2)	
Ex-smoker	23,935 (9.3)	12,366 (17.8)	7603 (18.9)	
Current smoker	6433 (21.9)	28,534 (41.1)	20,942 (52.0)	
Alcohol (g/week)	54.7 ± 110.5	109.3 ± 163.6	162.4 ± 205.4	<0.001
Activity (met-min/week)	379.6 ± 376.8	384.3 ± 381.1	371.4 ± 368.7	<0.001
AST (U/L)	21.7 ± 12.4	27.2 ± 43.2	35.7 ± 52.9	<0.001
ALT (U/L)	18.3 ± 14.5	30.9 ± 46.2	47.8 ± 63.1	<0.001
GGT (U/L)	20.6 ± 14.6	46.7 ± 15.9	93.5 ± 96.5	<0.001
Hemoglobin (g/dL)	13.6 ± 1.6	14.8 ± 1.5	15.3 ± 1.4	<0.001
Fasting glucose (mg/dL)	91.5 ± 15.0	98.2 ± 23.2	104.0 ± 30.2	<0.001
Total cholesterol (mg/dL)	186.7 ± 36.5	204.1 ± 39.5	214.8 ± 44.6	<0.001
Triglyceride (mg/dL)	89.3 ± 44.5	164.8 ± 80.7	258.7 ± 188.6	<0.001
HDL (mg/dL)	60.1 ± 21.4	52.5 ± 27.7	50.6 ± 32.3	<0.001
LDL (mg/dL)	113.6 ± 172.8	123.0 ± 108.9	120.0 ± 99.8	<0.001
Creatinine (mg/dL)	0.9 ± 1.1	1.1 ± 1.2	1.1 ± 1.3	<0.001

Data are expressed as numbers (%) unless otherwise indicated. Continuous variables are presented as mean ± standard deviation (SD). Abbreviations: FLI, fatty liver index; BMI, body mass index; SBP, systolic blood pressure; DBP, diastolic blood pressure; AST, aspartate aminotransferase; ALT, alanine aminotransferase; GGT, gamma glutamyl transferase; HDL, high-density lipoprotein; LDL, low-density lipoprotein.

**Table 2:** Association between FLI and incidence of lung abscess and CAP.

			Univariate Analysis			Multivariate Analysis					
						Model 1 *			Model 2 ^†^		
	Subjects	Event (%)	HR	95% CI	*p*	HR	95% CI	*p*	HR	95% CI	*p*
Lung abscess											
0 ≤ FLI < 30	258,203	266 (0.10)	Reference			Reference			Reference		
30 ≤ FLI < 60	69,429	116 (0.16)	1.58	1.27–1.96	<0.001	1.00	0.80–1.25	0.983	1.26	0.95–1.68	0.115
FLI ≥ 60	40,298	73 (0.18)	1.77	1.37–2.29	<0.001	1.23	0.94–1.61	0.129	1.67	1.06–2.63	0.028
CAP											
0 ≤ FLI < 30	258,203	31,988 (12.4)	Reference			Reference			Reference		
30 ≤ FLI < 60	69,429	8510 (12.3)	0.96	0.94–0.98	<0.001	0.97	0.94–0.99	0.022	1.06	1.02–1.09	<0.001
FLI ≥ 60	40,298	4436 (11.0)	0.89	0.86–0.91	<0.001	0.97	0.93–0.99	0.045	1.13	1.07–1.18	<0.001

* Model 1 was analysed with covariates including age and male sex. ^†^ Model 2 for lung abscess was analysed with covariates including age, male sex, systolic blood pressure (SBP), diastolic blood pressure (DBP) smoking, alcohol consumption, activity level, aspartate aminotransferase (AST), alanine aminotransferase (ALT), haemoglobin, and total cholesterol.

## Data Availability

The NHIS-NSC 2.2 is accessible to any researcher whose protocol is approved by the NHIS review committee “https://nhiss.nhis.or.kr (accessed on 16 April 2024)”. However, the datasets presented in this article are not readily available because the data access period from the National Health Insurance Service has expired. Requests to access the modified and saved datasets should be directed to the corresponding author. The original contributions presented in this study are included in the article and its supplementary material.

## Supplementary Materials

The following supporting information can be downloaded at: https://www.mdpi.com/article/10.3390/jcm14020542/s1, Table S1: Diagnosis defined by the 10th revision of the International Classification of Disease codes; Table S2: Disease-related healthcare utilization of lung abscess and CAP according to the FLI groups; Table S3: Univariate analysis of the association between clinical parameters and lung abscess and CAP.
