# Supplementary material for: Nonalcoholic Fatty Liver Disease Increases the Risk of Lung Abscess: Findings from a Nationwide Cohort Study
Source: J Clin Med. 2025 Jan 16;14(2):542. doi: 10.3390/jcm14020542 (PMC11765748; doi:10.3390/jcm14020542)
Supplement: Supplementary file 1 [file jcm-14-00542-s001.zip › jcm-3372091-supplementary.pdf]

Table S1. Diagnosed defined by the 10th revision of the International Classification of Disease codes.

	International Classification of Disease, 10th revision (ICD-10) code
Lung abscess	J85.x-J86.x
Community-acquired pneumonia	J10.x-J18.x
Autoimmune disease	M05.x-M09.x, M30.x-M36.x, M45.x
Chronic kidney disease	N18
Chronic liver disease	B15.x-B19.x, K70.x-K77.x, K80.x-K87.x, I85.x, I86.4, I98.2, I98.3, Z94.4
Chronic pulmonary disease	
Asthma	J45-046
Chronic obstructive lung disease	J41-44
Bronchiectasis	J47
Other interstitial lung diseases	J84
Rheumatoid lung disease	J99
Sarcoidosis	D86
Cerebrovascular disease	I60-I64
Immune deficiency disease	D80.x-D89.x/ HIV B20-24
Malignant disease	C00-C34, C39-C99, D00-D09
Neuromuscular disease	
Dementia	F00−F03, G30−G32
Parkinsonism	G20-21

Table S2. Disease-related healthcare utilization of lung abscess and CAP according to the FLI groups.

	Lung abscess				CAP			
	0≤FLI<30 (n=266 [58.5])	30≤FLI<60 (n=116 [25.5])	FLI≥60 (n=73 [16.0])	*P*	0≤FLI<30 (n=31,988 [71.2])	30≤FLI<60 (n=8,510 [18.9])	FLI≥60 (n=4,436 [9.9])	*P*
Hospitalizations	140 (52.6)	73 (62.9)	43 (58.9)	0.155	4,136 (12.9)	1,286 (15.1)	621 (14.0)	0.001
Incidence rate*	122.8	148.2	121.6	0.003	29.7	34.4	32.5	0.001
length of stay(days)	20.5 ± 12.2	22.7 ± 14.7	25.6 ± 16	0.030	17 ± 13	18.7 ± 15	18.3 ± 13	<0.001
ICU admission	1 (0.4)	0	0	1.000	5 (0.02)	3 (0.04)	0	0.411
Incidence rate*	0.88	0	0	NA	0.08	0.04	0	1.000
Mortality at 30 days	7 (2.6)	2 (1.7)	1 (1.4)	0.45	264 (0.8)	78 (0.9)	48 (1.1)	0.074

*per 1000-person year.

Data are expressed as numbers (%) unless otherwise indicated. Continuous variables are presented as mean ± standard deviation (SD). Abbreviations: FLI, fatty liver index; CAP, community-acquired pneumonia; ICU, intensive care unit.

Table S3. Univariate analysis of the association between clinical parameters and lung abscess and CAP.

	Lung abscess		CAP	
Variables	HR (95% CI)	*P*	HR (95% CI)	*P*
Age (years)	1.070 (1.063-1.077)	<0.001	1.021 (1.020-1.022)	<0.001
Male	3.062 (2.457-3.817)	<0.001	0.779 (0.765-0.794)	<0.001
Height(cm)	1.006 (0.996-1.016)	0.265	0.981 (0.980-0.982)	<0.001
Weight(kg)	0.997 (0.989-1.005)	0.435	0.988 (0.988-0.989)	<0.001
BMI(kg/m2)	0.975 (0.948-1.004)	0.090	0.986 (0.983-0.988)	<0.001
Waist circumference(cm)	1.006 (1.004-1.008)	<0.001	0.999 (0.998-1.000)	0.011
SBP(mmHg)	1.018 (1.013-1.024)	<0.001	0.999 (0.998-0.999)	0.001
DBP(mmHg)	1.022 (1.013-1.030)	<0.001	0.996 (0.996-0.997)	<0.001
Smoking				
Ex smoking	1.814 (1.375-2.938)	<0.001	0.928 (0.901-0.955)	<0.001
Current smoking	2.289 (1.875-2.795)	<0.001	0.819 (0.802-0.838)	<0.001
Alcohol(g/week)	1.001 (1.001-1.002)	<0.001	0.999 (0.999-1.000)	<0.001
Activity(met-min/week)	1.000 (0.999-1.000)	0.033	1.000 (1.000-1.000)	<0.001
AST(U/L)	1.001 (1.000-1.001)	0.009	1.000 (1.000-1.000)	0.028
ALT(U/L)	1.001 (1.000-1.001)	0.068	0.999 (0.998-0.999)	<0.001
GGT(U/L)	1.004 (1.003-1.005)	<0.001	1.000 (1.000-1.000)	0.132
Hemoglobin(g/dL)	1.075 (1.016–1.137)	<0.001	0.925 (0.920-0.930)	<0.001
Fasting glucose(mg/dL)	1.006 (1.004–1.008)	<0.001	1.000 (1.000-1.001)	0.049
Total cholesterol(mg/dL)	1.000 (0.098–1.002)	0.987	1.000 (1.000-1.000)	0.293
Triglyceride(mg/dL)	1.001 (1.000–1.001)	<0.001	1.000 (1.000-1.000)	0.057
HDL(mg/dL)	0.990 (0.983–0.996)	0.003	1.000 (1.000-1.000)	0.605
LDL(mg/dL)	0.999 (0.997–1.001)	0.373	1.000 (1.000-1.000)	0.078
Creatinine(mg/dL)	1.029 (0.983–1.077)	0.223	0.981 (0.972-0.990)	<0.001

Data are presented as mean ± SD or number (%), Abbreviations: CAP, community acquired pneumonia; BMI, body mass index; SBP, systolic blood pressure; DBP, diastolic blood pressure; Non, non-smoker; Ex, ex-smoker; AST, aspartate aminotransferase; ALT, alanine aminotransferase; GGT, gamma-glutamyl transferase; HDL, high density lipoprotein; LDL, low density lipoprotein
